# The life cycle impact for platinum group metals and lithium to 2070 via surplus cost potential

**DOI:** 10.1007/s11367-017-1329-4

**Published:** 2017-05-11

**Authors:** Dominik Jasiński, James Meredith, Kerry Kirwan

**Affiliations:** 1 Warwick Manufacturing Group, University of Warwick, Coventry, CV4 7AL UK; 2 Department of Mechanical Engineering, University of Sheffield, Sheffield, S1 3JD UK

**Keywords:** Life cycle impact assessment, Marginal cost increase, Metal scarcity, Minerals cost-cumulative availability curves, Resource depletion, Surplus cost

## Abstract

**Purpose:**

A surplus cost potential (SCP) indicator has been developed as a measure of resource scarcity in the life cycle impact assessment (LCIA) context. To date, quality SCP estimates for other minerals than fossils are either not yet available or suffer methodological and data limitations. This paper overcomes these limitations and demonstrate how SCP estimates for metals can be calculated without the utilisation of ore grade function and by collecting primary economic and geological data.

**Methods:**

Data were collected in line with the geographical distribution, mine type, deposit type and production volumes and total production costs in order to construct cost-cumulative availability curves for platinum group metals (PGMs) and lithium. These curves capture the total amount of known mineral resources that can be recovered profitably at various prices from different types of mineral deposits under current conditions (this is, current technology, prevailing labour and other input prices). They served as a basis for modelling the marginal cost increase, a necessary parameter for estimating the SCP indicator. Surplus costs were calculated for different scenario projections for future mineral production considering future market dynamics, recyclability rates, demand-side technological developments and economic growth and by applying declining social discount rate.

**Results and discussion:**

Surplus costs were calculated for three mineral production scenarios, ranging from (US$_2014_/kg) 6545–8354 for platinum, 3583–4573 for palladium, 8281–10,569 for rhodium, 513–655 for ruthenium, 3201–4086 for iridium and 1.70–5.80 for lithium. Compared with the current production costs, the results indicate that problematic price increases of lithium are unlikely if the latest technological trends in the automotive sector will continue up to 2070. Surplus costs for PGMs are approximately one-third of the current production costs in all scenarios; hence, a threat of their price increases by 2070 will largely depend on the discovery of new deposits and the ability of new technologies to push these costs down over time. This also applies to lithium if the increasing electrification of road transport will continue up to 2070.

**Conclusions:**

This study provides useful insight into the availability of PGMs and lithium up to 2070. It proves that if time and resources permit, reliable surplus cost estimates can be calculated, at least in the short-run, based on the construction of one’s own curves with the level of quality comparable to expert-driven consulting services. Modelling and incorporating unknown deposits and potential future mineral production costs into these curves is the subject of future work.

**Electronic supplementary material:**

The online version of this article (doi:10.1007/s11367-017-1329-4) contains supplementary material, which is available to authorized users.

## Introduction

Over the last two decades, several models for evaluating resource availability and assessing the potential of resource depletion have been proposed in the context of life cycle impact assessment (LCIA). These models can be classified into four major groups: (1) models aggregating resource consumption based on mass or energy (Huijbregts et al. 2010; Saurat and Ritthoff 2013), (2) models quantifying thermodynamic losses (e.g. exergy and solar energy) (Finnveden and Östlund 1997; Dewulf et al. 2007), (3) models measuring diminishing geological stock available within planetary boundaries (Guinée 1995; Hauschild and Wenzel 1998; Van Oers et al. 2002); and, (4) opportunity cost models measuring the sacrifice that society has to make in order to obtain an additional quantity of a given resource (Humphreys 2013; Drielsma et al. 2016), such as higher energy requirements (Müller-Wenk 1998; Goedkoop and Spriensma 2001; Jolliet et al. 2003) and the change in future marginal extraction costs resulting from the combination of depletion, exploration results and cost-reducing innovation (Steen 1999; Goedkoop et al. 2008; Ponsioen et al. 2014).

The first two groups of models are considered inadequate indicators of resource depletion because they address the consumption of natural resources as opposed to resource scarcity and declining availability (Lindeijer et al. 2002; Stewart and Weidema 2005; Rørbech et al. 2014). Although the third group of methods gives an indication of time frames as well as the rates at which minerals are depleted in line with present-day technology and demand, Tilton (2003) proved that the life expectancy of most minerals in the Earth’s crust will exceed millions, or even billions, of years. This suggests that the physical availability of minerals will never become a serious issue for mankind. Rather, it is more likely that the rising costs of extracting a mineral such as copper (Cu) from the Earth will eradicate demand for it a long time prior to exhaustion of the physical resource itself (Tilton and Lagos 2007; Humphreys, 2013). For this reason, a function of the long-run costs and prices of minerals provides a more promising early warning indicator of impending resource scarcity than do measures related to their physical availability (Yaksic and Tilton 2009; Humphreys 2013; Drielsma et al. 2016).

A surplus cost potential indicator, classified under the umbrella of opportunity cost methods, measures the net present value of the increase in mineral production costs associated with each additional extraction of a mineral commodity (Goedkoop et al. 2008; Ponsioen et al. 2014). A basic assumption of the method is that mining companies operate mainly to maximise profits and therefore higher quality and the least expensive deposits are mined first. This, however, is not always the case mainly because of the discovery of new deposits, cost-reducing technological innovations and other geopolitical factors such as trade barriers or strategic stocking of resources (Tilton 2003; Ponsioen et al. 2014; Drielsma et al. 2016). Nonetheless, the uniqueness and strength of this method lies in its ability to model resources that are produced as co-products by using a system of price allocation. Many minerals are almost exclusively mined as co-products of other metals (e.g. rhodium, ruthenium, iridium and rare earth elements), and surplus cost does more justice to the real world than methods that only address the depletion of single minerals (EC-JRC 2011; Hauschild et al. 2013). Surplus cost was selected by the EC-JRC (EC-JRC 2011) as showing promise and as the best of the existing measures available for capturing resource depletion at the endpoint level, although it is not yet considered sufficiently mature for recommendation.

To date, quality surplus cost estimates have been derived only for fossil fuels (oil, natural gas and coal) (see Ponsioen et al. 2014). These estimates were based on the actual data of mineral reserves and production costs published by relevant institutions, such as the International Energy Agency (IEA) and German Federal Institute for Geosciences and Natural Resources. Surplus cost estimates for other minerals are either not yet available or require the improvements. For example, the utilisation and linking of the ore grade decrease function with the increasing marginal extraction cost of metals in existing surplus cost studies has been heavily criticised by relevant organisations in the metals and minerals mining sector (e.g. the European Association of Mining Industries, the Nickel Institute and the European Copper Institute) (see Drielsma et al. 2016). Factors other than ore grades affect the cost of mineral extraction (e.g. mine type, new discoveries, labour cost and technological developments). Hence, minerals other than fossils (e.g. metals) could be modelled without an ore grade decrease function, something that in any case has been heavily criticised by authoritative bodies. Furthermore, production cost data pertaining to minerals other than fossils are much more difficult to obtain than, for example, geological data (Yaksic and Tilton 2009). Existing surplus cost studies for metals used a simplified approach by assuming constant mining costs across all mines (see Goedkoop et al. 2008). Vieira et al. (2016) adjusted surplus cost estimates for 12 metals by using the actual cost data purchased from the commercial database World Mine Cost Data Exchange. However, these data do not recognise the characteristics of different deposits and mining technologies. Also, these data are not publically available and it is therefore not possible to reproduce and validate existing surplus cost estimates.

The goal of this paper is to overcome these limitations and demonstrate how surplus cost estimates for metals, similarly to fossils, could be calculated without the utilisation of ore grade function and without the need to rely on authoritative institutions or purchase data from commercial databases. The metals selected in this study are platinum, palladium, rhodium, ruthenium and iridium, all of which are platinum group metals (PGMs). The results for PGMs will be compared with those for lithium, which is a potential substitute for PGMs in future automotive applications, depending on the rate and extent to which electric cars replace cars with internal combustion engines.

## Research methods

### The surplus cost potential indicator

The surplus cost potential indicator (SC_*x*_) is based on three parameters (Ponsioen et al. 2014), as evident in Eq. (1) and Eq. (2):

1 $$ {\mathrm{SC}}_x=\sum_{t=1}^T\left({{\mathrm{MCI}}_x}^{\ast }{P_{x, t}}^{\ast }\ \frac{1}{{\left(1+ d\right)}^t}\right) $$

where,

2 $$ {\mathrm{MCI}}_x=\frac{\varDelta {\mathrm{Cost}}_x}{\varDelta {P}_x} $$

MCI_*x*_ is the marginal cost increase of mineral *x* expressed as a ratio of the change in the cost per kilogramme of mineral *x* (∆Cost_*x*_) to the change in the amount to be produced in the future (∆*P*_*x*_). *P*_*x,t*_ is the annual production of mineral *x* in year *t* counting from the base year, *T* is the year in which the considered mineral resource *x* is depleted and *d* is the discount rate. The process of modelling and estimating these parameters is explained in the following subsections.

### The construction of cost-cumulative availability curves

**Fig. 1:**
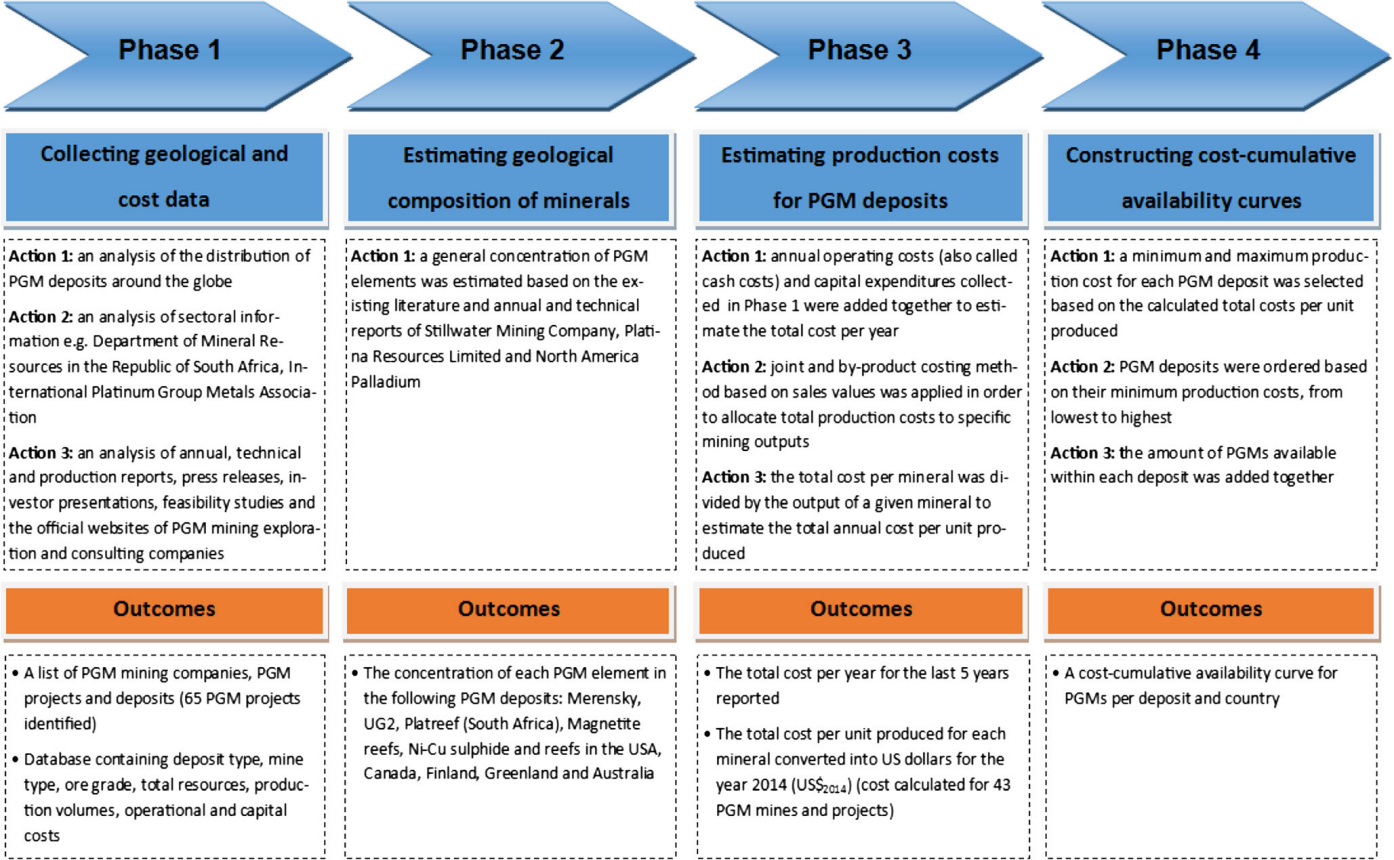
The process of constructing cost-cumulative curves for PGMs

The MCI parameter can be derived from the function of cumulative mineral production and difference in its production costs (Ponsioen et al. 2014). This requires the construction of cost-cumulative availability curves which provide geological knowledge of existing mineral deposits, their sizes as well as potential costs at which these deposits could be extracted. Data for constructing these curves is available for lithium (see Yaksic and Tilton, 2009), but not for PGMs. The development process of the cost-cumulative availability curve for PGMs is summarised in Fig. 1, and is explained as follow:

- *Phase 1*: involved collecting and analysing data about the distribution of known PGM deposits, mining companies and projects around the globe as well as data on deposit types, mine types, ore grades, total resources, production volumes, operational and capital costs for PGM mines and deposits. The best data for phase 1 were collected from the US Geological Survey (USGS 2014), British Geological Survey (BGS 2009), Geoscience Australia (Hoatson et al. 2014), Natural Resources Canada (NRC 2015), Geological Survey of Finland (online), Johnson Matthey (2013b), Department of Mineral Resources in the Republic of South Africa (Moumakwa 2014), International Platinum Group Association and a number of annual, technical and production reports, press releases, investor presentations, feasibility studies and official websites of PGM mining, exploration and consulting companies.
- *Phase 2*: involved estimating a geological composition of PGMs depending on the deposit types. A typical deposit contains various metals but there is usually a main metal that justifies the exploitation of a given deposit (Vieira et al. 2016). PGMs are mined as both the main and accompanying metals of nickel (Ni) and copper deposits (Hagelüken and Meskers 2010). A general concentration of PGM elements was assumed based on the existing literature (Theart and De Nooy 2001; Crundwell et al. 2011; Zientek et al. 2014) and the websites and reports of mining and exploration companies such as Stillwater Mining Company, Platina Resources Limited and North America Palladium.
- *Phase 3*: annual operating costs (also called cash costs), capital expenditures and production volumes were collected from a variety of sources (see the data collection section, phase 1). The estimation of capital expenditures on an annual basis is more complicated than for operational costs since capital expenditures are incurred largely at the start of mining, with additional irregular expenditures incurred in subsequent years of running the project. If ultimate capital expenditures for the lifetime of the project are not known, the annual distribution of capital costs is best reflected by the depreciation of buildings and equipment (Aguilera et al. 2009). This approach was also adopted in this study. An accounting technique of joint and by-product costing based on sale values had to be applied in order to allocate total production costs to specific mining outputs (Drury 2013). All costs were adjusted for inflation using the CPI inflation calculator available at the Bureau of Labor Statistics website and converted into US dollars for the year 2014 ($US_2014_).
- *Phase 4*: following the estimation of geological distribution and production costs, the cost-cumulative availability curve for PGM deposits and countries was then constructed following a process similar to that used by Yaksic and Tilton (2009) for lithium. For each deposit, a minimum and maximum production cost was selected based on the calculated total costs per unit produced. This selection of minima and maxima enabled the dynamics and uncertainties associated with the potential fluctuation of PGM production costs in the future to be captured. It also allowed for the inclusion in the curve of those known projects (deposits) for which production costs could not be calculated, assuming they had the potential to be mined within the cost range estimated for other deposits in the country that had similar geological configurations, geographical locations and socio-economic situations. The cost-cumulative availability curve was created by ordering PGM deposits based on their minimum production costs, from lowest to highest, and adding together the amount of PGMs available within each deposit.

Each phase of constructing the cost-cumulative availability curve for PGMs as well as underlying data is explained in details in supplementary information. A similar curve per deposit type and country was constructed for lithium based on data published by Yaksic and Tilton (2009).

### Modelling the MCI parameter

In order to develop a cumulative production slope representing the MCI parameter from the minimum and maximum costs per deposit type of PGMs and lithium, Ponsioen et al. (2014) proposed a statistical Monte Carlo technique, a simulation method that relies on repeated, multiple and random trials and statistical analysis in order to determine the expected value from a probable distribution of values (Barreto and Howland 2005; Raychaudhuri 2008; Korn et al. 2010). Assuming that the mineral production cost per deposit is a random number between the minima and maxima, the Monte Carlo method enables generation of the expected value with a specified level of certainty. There are a number of different software packages for use with the Monte Carlo method; however, Monte Carlo simulations can also be performed using Microsoft Excel spreadsheets (Barreto and Howland 2005; Raychaudhuri 2008), as was the case in this study. The process employed in running Monte Carlo simulations was as follows:

- The total known resources (assumed to be equal to proved and probable reserves and measured, indicated and inferred mineral resources) available for each mineral were divided into equal production intervals for each mineral, respectively 10,000 kg for platinum, 10,000 kg for palladium, 5000 kg for ruthenium, 1200 kg for rhodium, 1000 kg for iridium and 2E + 10 kg for lithium from oceans and 2E + 7 kg for other lithium deposits.
- A range of production costs was allocated to each interval based on the cumulative availability and production cost per deposit.
- Assuming a uniform distribution of production costs between the minima and maxima, random values for each production interval were generated using the *RAND()* function in Excel.
- In order to obtain an accurate value, the Monte Carlo method is based on a large number of simulations. The higher the number of simulations, the more accurate the results that can be obtained; however, the number of simulations is not that critical provided confidence bounds are also computed (Korn et al. 2010). Similar to the process used by Ponsioen et al. (2014), 10,000 simulations were run for each production interval, the minimum for industry standards (Field 2009).

Cost values obtained from the Monte Carlo simulations were ordered from the lowest to highest and the cost-cumulative production curve was developed with the range for each production interval and the mean slope representing the MCI of each mineral. Further statistical analysis was conducted, including estimation of the median, standard deviation and confidence bounds in order to estimate the precision of the obtained values (Raychaudhuri, 2008).

### Future mineral production

One of the challenges in estimating the surplus cost indicator is predicting how the production of a given mineral will change over time. Scenario analysis is a critical tool in the world of finance and economics and is used to determine and analyse events that may take place in the future (Ringland and Schwartz 1998; Van der Heijden 2011).

Three different scenarios for the future production of PGMs and lithium were developed. Common conditions for all three scenarios, such as future population and economic growth, demand for minerals from non-automotive uses and recyclability and mineral loadings per vehicle, are summarised in Table 1.

**Table 1:** PGM and lithium demand forecast assumptions to 2070

	Baseline scenario, Blue map scenario and Blue map without FCVs scenario	References
Timespan, region	2070, global	(Taylor 2010); (Tanaka 2011); (Johnson Matthey 2013b)
Economic conditions	Global gross domestic product grows by an average of 3.1%.	
Social conditions	The world’s population will grow by an average of 0.7%, reaching 9.2 bn in 2050 and 10.5 bn in 2070.	
Automotive market	Annual vehicle catalyst and battery production will equal growth in the vehicle fleet (one catalyst and one battery per vehicle, in use for 160,000 km). Demand for LDVs will grow by an average of 2.5%, from 60.5 million in 2010 to 268 million in 2070 (see Fig. 1 for demand projections). Demand for MDVs and HDVs will grow by an average of 2%, from 4.5 million in 2010 to 14.7 million in 2070 (see Fig. 2 for demand projections).	
Other markets	Demand for PGMs from other sectors: • Jewellery, chemical, electrical, glass, other 1% growth until 2070. Demand for lithium from other sectors: • Secondary batteries (rechargeable and portable devices) 10% growth until 2020, 3% growth until 2050 and 1% growth after 2050; • Primary batteries (non-rechargeable devices) 5% growth until 2020, 3% growth until 2050 and 1% growth after 2050; • Lubricating greases 3% growth until 2030, 1% after 2030; • Ceramic and glass 2% growth until 2030, 0.5% after 2030; • Air conditioning 3% growth until 2020, 1% after 2020; • Aluminium 5% reduction until 2020, no lithium consumption after 2020; • Others 2% growth until 2020, 1% after 2020.	(Yaksic and Tilton 2009); (Johnson Matthey 2013a).
Recyclability	Recycling can reduce primary metal consumption through the use of secondary materials. There are 2 major measures of recyclability: recycling rate and recycled content. The recycling rate measures the amount of metal recycled from scrap. Recycled content is defined as the annual tonnage of material scrap consumed divided by tonnage of material produced, depending on how much scrap is available. Hence, material content is a better measure of recyclability if one wishes to understand primary metal consumption based on existing recycling rates. Even with a high recycling rate, the amount of recycled content can be low due to a low amount of available material scrap. For this reason, recycled content was used in this study as a measure of reduced primary metal consumption as a result of recycling activities. The recycled content of PGMs is between 10 and 50%, with an average of 24% between 2008 and 2013. The level of recycled content will grow by an average of 1.5% until it reaches 90%. The recycled content of lithium is currently below 1%. This is expected to grow with the increased use of Li-ion batteries in EVs and HVs. Growth is assumed at an average rate of 2.7% until the amount of recycled content reaches 80%.	(Yaksic and Tilton 2009); (Graedel et al. 2011); (Johnson Matthey 2013a); (Schneider et al. 2014)
Mineral loadings per vehicle	PGM loading per vehicle is the average between the US and European emissions standards and is assumed to decrease over time. The average PGM loadings (grammes per vehicle) for LDVs are as follows: • Petrol 3.52 until 2030, 3.3 until 2050 and 2.64 after 2050 • Diesel 7.25 until 2030, 6.9 until 2050 and 5.66 after 2050 • Hybrid/PHEVs 2.7 until 2030, 2.6 until 2050 and 2.07 after 2050 • FCVs 16 until 2030 and 8 after 2030 Larger engines require more PGMs, therefore the average PGM loadings for MDVs and HDVs were doubled and are as follows: • Petrol 7.03 until 2030, 6.6 until 2050 and 5.28 after 2050 • Diesel 7.25 until 2030, 6.9 until 2050 and 5.66 after 2050 • Advanced biofuels/CTL/GTL/Natural gas 5.38 until 2030, 5.1 until 2050 and 4.15 after 2050 Average lithium loading was assumed to be 140 g/kWh with EVs needing on average a 42-kW battery (60 kW for electric light trucks), PHEVs a 7.5-kW battery and hybrids a 1.2-kW battery.	(Bloxham 2009); (Lowe et al. 2010); (Sun et al. 2011); (Goonan 2012); (Cooper and Beecham 2013); (Nguyen et al. 2014)

**Fig. 2:**
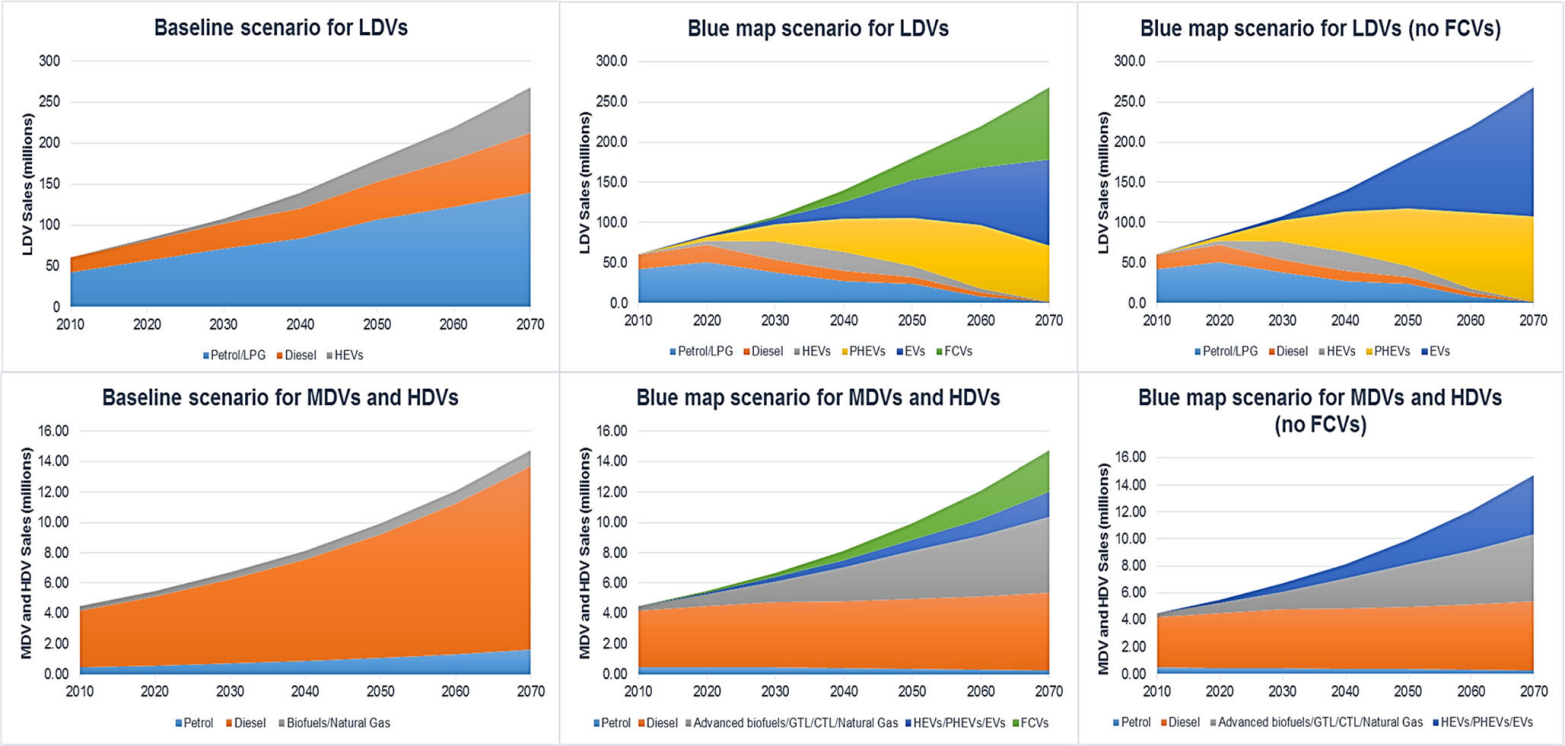
Projections of future demand for vehicles in three different scenarios (adapted from Taylor, 2010 and Tanaka, 2011)

A major factor set to influence demand for PGMs and lithium over the coming decades is the use of whichever energy source becomes dominant for road transport. For example, the automotive sector’s use of PGMs should decline and demand for lithium increase if, or once, electric cars replace cars with internal combustion engines. On the other hand, current automotive fuel cells rely heavily on platinum-coated catalytic converters, meaning any penetration of fuel cell technology will have a severe impact on demand for PGMs, with one fuel cell vehicle (FCV) requiring approximately 30 g of platinum (Sun et al. 2011). The projected demand for light-duty vehicles (LDVs), medium-duty vehicles (MDVs) and heavy-duty vehicles (HDVs) in three different energy technology penetration scenarios was based on two IEA reports; Energy Technology Perspectives (Taylor 2010) and Technology Roadmap: Electric and Plug-in Hybrid Electric Vehicles (Tanaka 2011). These projections are presented in Fig. 2e and are explained as follows:

- *Scenario 1* (*baseline*): in the baseline scenario, existing trends will continue and petrol and diesel vehicles will be dominant in the future with only a small proportion of hybrid vehicles (HVs) for LDVs and natural gas biofuels for MDVs and HGVs.
- *Scenario 2* (*Blue map*): the Blue map scenario assumes a 50% global reduction in greenhouse gas (GHG) emissions by 2050 relative to their 2000 level as the result of a strong mix of policy instruments focusing on climate change. Obtaining the maximum efficiency gains in reducing GHG emissions will require a large penetration of plug-in hybrid electric vehicles (PHEVs), fully electric vehicles (EVs) and FCVs for LDVs. Electrification and fuel cells are also assumed for MDVs, along with an increase in the use of alternative fuels in HDVs, in particular advanced biofuels, gas-to-liquid, coal-to-liquid and natural gas.
- *Scenario 3* (*Blue map without FCVs*): the third scenario is based on the same assumptions as scenario 2, with the difference that the automotive sector will shift towards full electrification without penetration of fuel cell technology. Hydrogen fuel cell systems have the potential to be a clean and efficient power option for vehicles, but also have many technical and economic challenges still to overcome prior to their full commercialisation (Sun et al. 2011).

### Selecting a discount rate

The selection of an appropriate discount rate is based on value choices and is therefore subjective. Environmental economists are far from a consensus on which discount rate to apply (Kahn and Greene 2013). For example, Ponsioen et al. (2014) used three discount rates (0, 3 and 15%) to estimate surplus cost for fossils. The ReCiPe method used a 2–5% range of discount rates (Goedkoop et al. 2008). The World Bank utilised a 4% discount rate to estimate the snatural capital in their wealth accounts (Jarvis et al. 2011). The UN System of Environmental-Economic Accounts (2012) recognises two types of discount rate: individual and social. Individual discount rates consider the preferences and perspectives of an individual consumer or company and the likelihood of them earning interest. Social discount rates consider time and risk for society as a whole, and societies, unlike individuals, must give greater consideration to the interests of future generations. For this reason, social discount rates are usually lower than individual discount rates. Private companies tend to use discount rates higher than 10%, while social discount rates are usually below 5% (Ponsioen et al. 2014).

The UK Office for National Statistics recommends a third option of choosing a uniform 3.5% social discount rate, to be used for all types of natural assets regardless of the purpose of the exercise (Kahn and Greene 2013). This option was first outlined in HM Treasury’s Green Book for use by UK authorities following consultation between experts and government officials. It has since been adopted by the French authorities and is also considered by US officials for all sustainability projects (Cropper et al. 2014). This option uses a declining uniform discount rate for impacts assessed over the very long term, at the rates presented in Table 2.

**Table 2:** Declining social discount rate proposed by HM Treasury (2003)

Number of years	0–30	31–75	76–125	126–200	201–300	300+
Discount rate	3.5%	3%	2.5%	2%	1.5%	1%

Declining long-term discount rates better represent the distribution of uncertain levels of economic growth into the distant future, or times when growth is unevenly distributed over time. Since natural resources are of long-term value to society, it makes sense to use the declining social discount rate for the purpose of this study.

## Results

### Cost-cumulative availability of PGMs and lithium

**Fig. 3:**
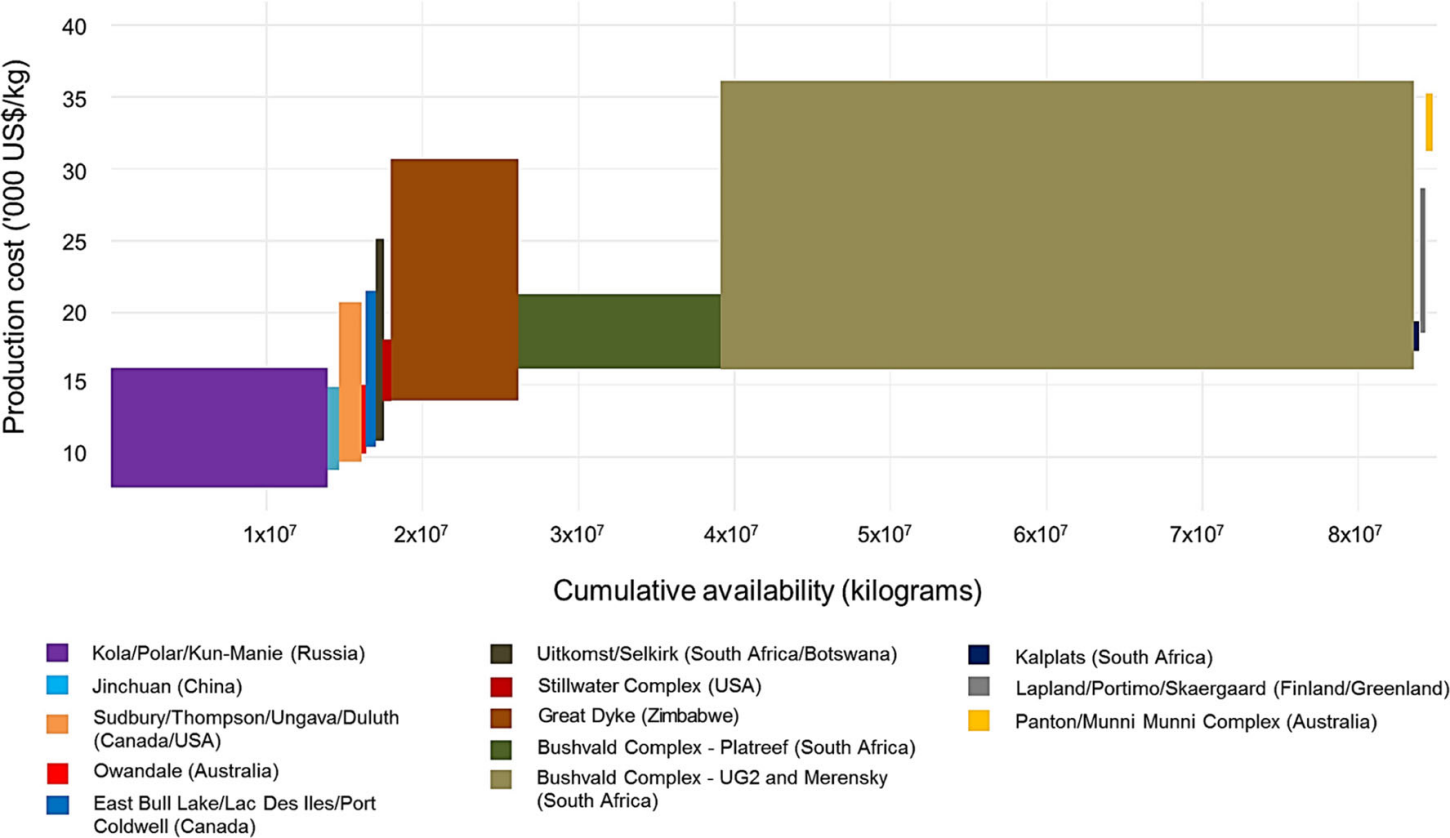
Cost-cumulative availability of PGMs per deposit

PGMs occur in a wide variety of geological settings and are derived from deposits of several types, with two major deposit groups being: platinum group element (PGE)-dominant deposits (Merensky, UG2, Platreef and the dunite pipes) and Ni-Cu-dominant deposits (see BGS 2009). In PGE-dominant deposits, PGMs are the dominant economic components, with Ni and Cu as minor by-products. Ni-Cu-dominant deposits are the most important sources of nickel worldwide. Copper, cobalt (Co), PGEs (primarily palladium), gold and sometimes silver (Ag) and chromium (Cr) are mined as accompanying metals (BGS 2009). Of the 65 PGM projects examined, the total cost per unit produced was estimated for 43. Based on these estimates, a minimum and maximum cost was allocated to each deposit and the cost-cumulative availability curve for PGMs was constructed as shown in Fig. 3. The underlying data used for construction of the CAC, including a list of known PGM deposits, along with estimates of their quantities and production costs, are provided in Table S3 and S4 in the Electronic Supplementary Material.

Accompanying metals usually contribute very little to the total revenue of a mining company and therefore the production cost of PGMs from Ni-Cu-dominant deposits (mostly in Russia, China and Canada) is lower than in the case of PGE-dominant deposits. Also, the production cost of PGMs from Ni-Cu-dominant deposits depends largely on the production costs and market values of Ni and Cu rather than the PGMs themselves. For example, an increase in the production costs and a decrease in the prices of Ni and Cu will shift the segment of the CAC pertaining to Ni-Cu-dominant deposits up despite there being no change or disruption to the PGM market. However, deposits where PGMs are only a by-product still make a significant contribution to the total availability of PGMs and were therefore included in Fig. 3.

To date, lithium has primarily been extracted, in all parts of the world, from two types of resources—brines and minerals (spodumene, lepidolite, petalite, amblygonite and eucriptite). Brines are currently the least expensive (no mining is required) and most relevant source of lithium. In addition to brines and mineral deposits, lithium can also be obtained from clays (hectorite) and seawater, both of which are potential future sources. The major global producers are Chile, Australia, Argentina, China and the USA, accounting for over 90% of the total production (Yaksic and Tilton 2009). Lithium, the same as PGMs, is mined both as a dominant metal (mainly from Li-rich pegmatites, which also contain other metals such as tin and beryllium) and as a by-product of other elements, mainly potash (K) (Nassar et al. 2015).

**Fig. 4:**
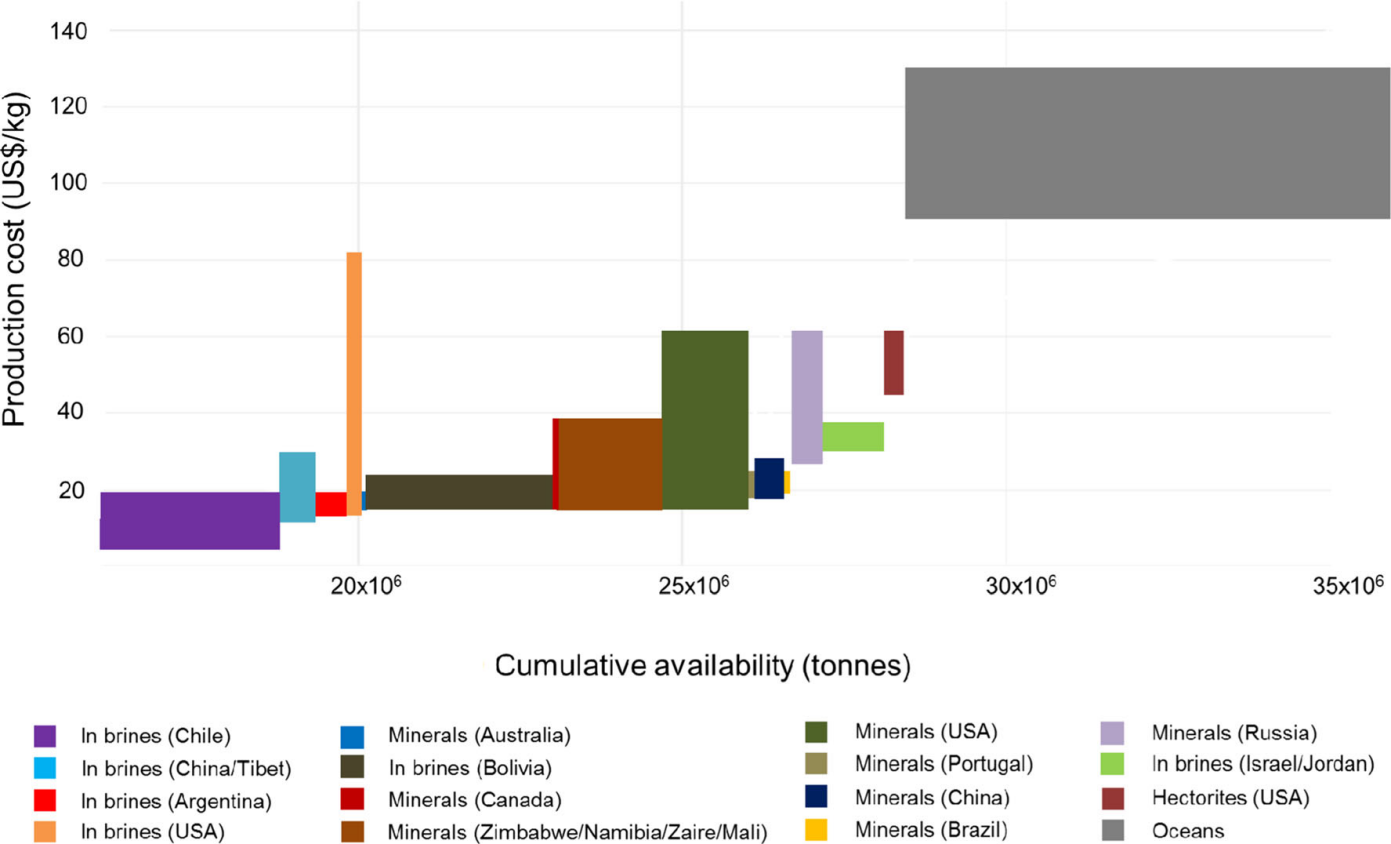
Cost-cumulative availability of lithium per deposit

The cost-cumulative availability of lithium is presented in Fig. 4 and the underlying data used to construct the curve are given in Table S5 in the Electronic Supplementary Material. The original production cost data (vertical axis) in Yaksic and Tilton (2009) were given in dollars per pound of lithium carbonate and available resources were measured in tonnes of contained lithium. In order to eliminate this difference in units, production cost data were converted into dollars per kilogramme of contained lithium by assuming that 5.323 kg of lithium carbonate contains 1 kg of lithium metal. Production costs were then adjusted for inflation to convert data from 2009 to 2014. The horizontal axis shows available resources after processing losses and assumes recovery rates of 50% for minerals, 50% for hectorites, 45% rate for brines and 20% for oceans.

Figure 4 is incomplete as it contains only a small proportion of the lithium available from seawater (oceans). This is because the amount of lithium recoverable from the oceans is vast, 44.8 billion tonnes, and it was not possible to fit this into the graph with the lithium deposits. This does not pose a serious problem provided we keep in mind there is an almost infinite supply of lithium from seawater (see Table S5 in the Electronic Supplementary Material for the specific amounts of lithium recoverable from each deposit).

### MCI results for PGMs and lithium

**Fig. 5:**
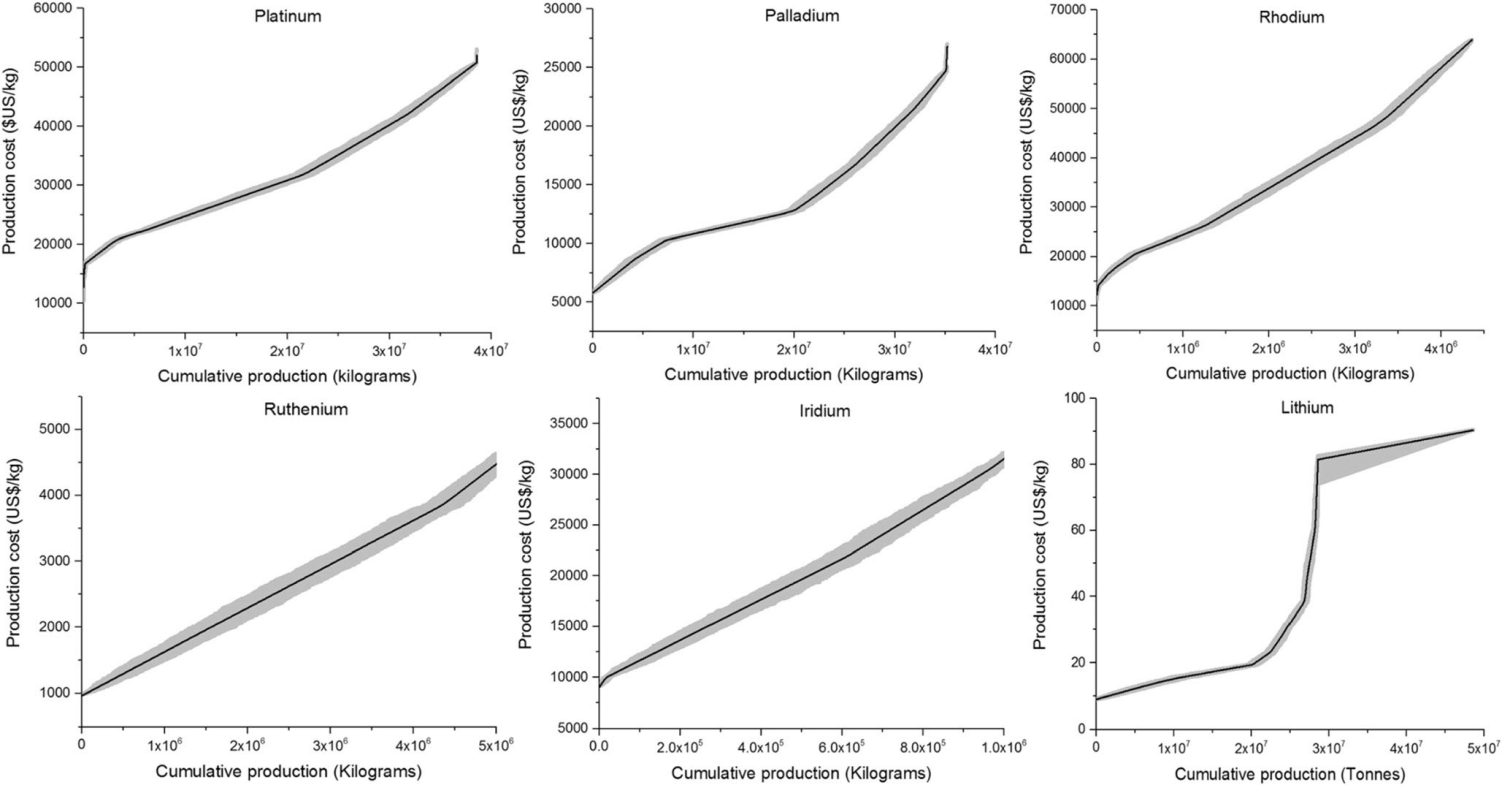
Cost-cumulative production curves (mean slope) for each mineral derived based on Monte Carlo simulations with the *grey area* representing the cost range

A statistical overview of the Monte Carlo simulations and MCI results obtained for each PGM and for lithium are presented in Table 3. The results represent the average MCI per mineral with 95% confidence bounds. The average difference between the mean and median for all metals is 0.45%, suggesting the data are normally distributed (Levin and Rubin 1998). Individual cost-cumulative production curves for each metal, with the mean slope and cost range per 10,000 kg of platinum, 10,000 kg of palladium, 5000 kg of ruthenium, 1200 kg of rhodium, 1000 kg of iridium and 2 × 10^10^ kg of lithium from oceans and 2 × 10^7^ kg from other deposits, are given in Fig. 5. The slope for lithium is incomplete for the same reasons as in the case of the cost-cumulative availability graph. The cumulative production of each individual PGM was estimated based on the general concentration of PGM elements in different deposit types (see Table S1 in Electronic Supplementary Material). Production of ruthenium and iridium is only reported by companies operating at the Bushvald Complex in South Africa. Companies mining other deposits, including all Ni-Cu-dominant deposits, report production of only two or three major PGMs, including platinum, palladium and rhodium.

**Table 3:** Average MCI calculations in US$ per kilogramme of mineral produced

Metal	MCI (US$_2014_/kg)	Std. dev. (US$_2014_/kg)	95% confidence interval—lower boundary (US$_2014_/kg)	95% confidence interval—upper boundary (US$_2014_/kg)
Platinum	1.019 × 10^−3^	3.852 × 10^−5^	9.434 × 10^−4^	1.094 × 10^−3^
Palladium	5.967 × 10^−4^	5.889 × 10^−6^	5.852 × 10^−4^	6.082 × 10^−4^
Rhodium	1.186 × 10^−2^	1.144 × 10^−4^	1.164 × 10^−2^	1.208 × 10^−2^
Ruthenium	7.344 × 10^−4^	1.002 × 10^−6^	7.325 × 10^−4^	7.364 × 10^−4^
Iridium	2.292 × 10^−2^	6.344 × 10^−5^	2.280 × 10^−2^	2.305 × 10^−2^
Lithium	1.116 × 10^−9^	4.815 × 10^−11^	1.022 × 10^−9^	1.210 × 10^−9^

It is evident from Table 3 that the MCI estimates for the six metals are different, ranging from 2.292 × 10^−2^ for iridium to 1.116 × 10^−9^ for lithium. The reasons for this substantial difference are twofold. First, society places a higher value on PGMs than on lithium and is thus prepared to spend more to extract 1 kg of, for example, palladium than it is for the same quantity of lithium. Second, the available resources for lithium are incomparably higher than for PGMs.

### Surplus cost potential for PGMs and lithium

**Fig. 6:**
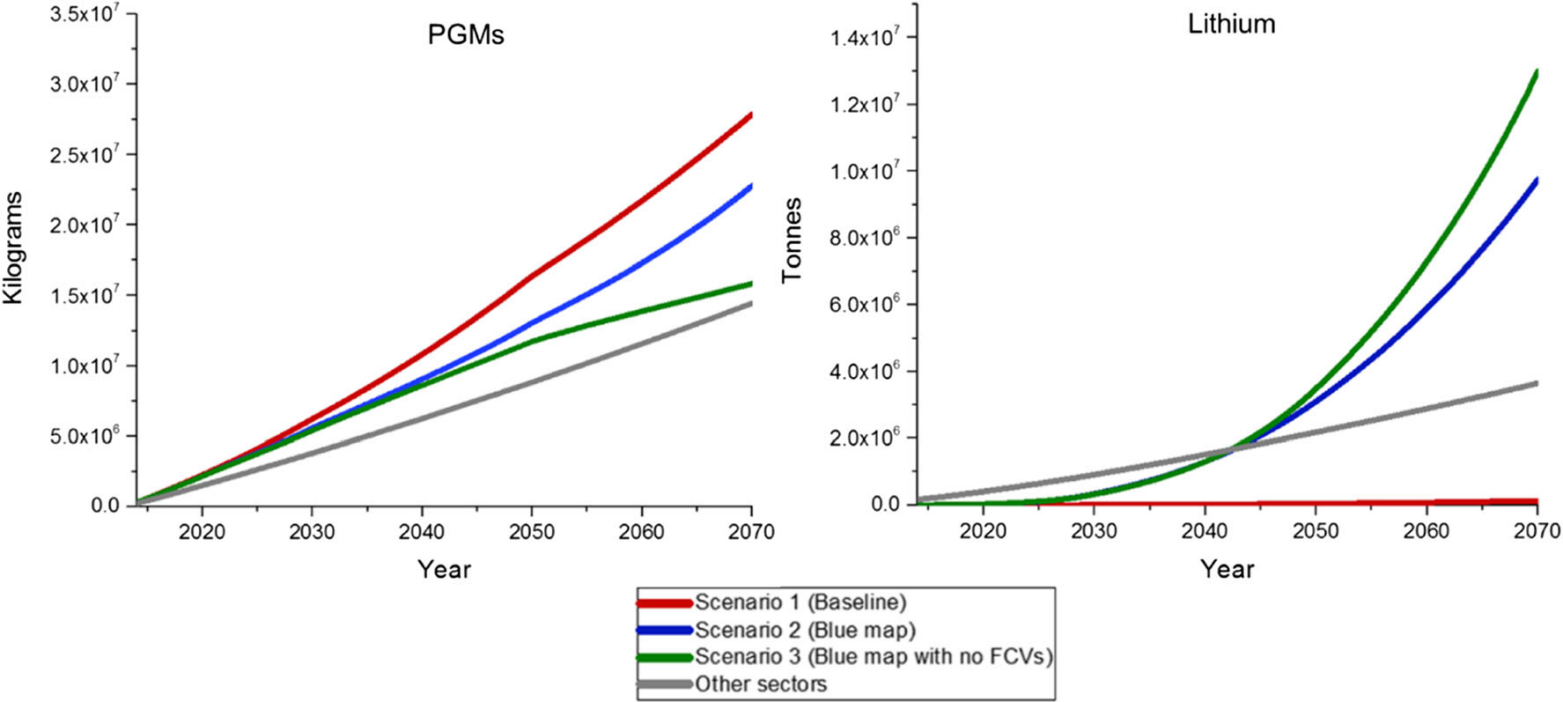
Cumulative supply of primary PGMs and lithium in the automotive sector based on three different scenarios, and in other sectors

The cumulative supplies, estimated as in Sun et al. (2011) (mineral demand adjusted to mineral supply from recycling), of primary PGMs and lithium based on scenario analysis (up to 2070) are presented in Fig. 6. These scenarios are somewhat optimistic, especially with regard to the rate at which the automotive sector shifts to full electrification and fuel cells.

As was evident in Fig. 6, supply of primary PGMs by 2070 is greatest in scenario 1, as in this case, diesel and petrol vehicles will continue to be dominant in the future. In scenarios 2 and 3, supply of PGMs will slacken after 2030 as PHEVs and EVs begin to replace cars with internal combustions engines. EVs do not require the use of PGMs, while PHEVs need significantly smaller quantities than petrol and diesel cars (see Table 1). In scenario 2, this trend will continue up to the point where FCVs become fully commercialised. With an increase in sales of FCVs, supply of PGMs will increase at a higher rate than in scenario 1 as these cars use more PGMs in their catalysts than internal combustion engines. Hence, after 2070, the cumulative supply of PGMs in scenario 2 should exceed the supply in scenario 1. Scenario 3 assumes that no FCVs will be used in the future due to technological limitations. For this reason, supply of PGMs in the automotive sector will systematically decrease as the world shifts towards full electrification. In fact, after 2070, supply of PGMs in the automotive sector will be lower than for other sectors. Use of PGMs in the automotive sector will continue, albeit at a lower rate, mainly for MDVs and HDVs, which will themselves progress towards electrification at a much slower pace than LDVs.

The cumulative supply scenarios for primary lithium are expected to work conversely to those for PGMs. In scenario 1, supply of lithium for other sectors will grow at a faster rate than supply for the automotive sector as no electrification of vehicles is assumed. In scenario 2, supply of lithium will grow substantially by 2050 before weakening in line with increasing sales of FCVs. The rate of growth in the supply of lithium will be at its greatest in scenario 3, once the road transport industry shifts to PHEVs and EVs.

Based on these future primary mineral supply scenarios and by applying the decreasing discounting rate, surplus cost estimates were calculated for PGMs and lithium, as presented in Table 4. These estimates were supplemented with the current average production costs for PGMs and lithium for comparison.

**Table 4:** Surplus cost potential for each mineral in three different production scenarios compared with the current average production costs

	Surplus cost US$_2014_/kg			Current average production costs US$_2014_/kg
Metal	Scenario 1 (baseline)	Scenario 2 (Blue map)	Scenario 3 (Blue map with no FCs)	
Platinum	8354	7428	6545	37,859
Palladium	4573	4066	3583	19,665
Rhodium	10,569	9398	8281	31,934
Ruthenium	655	583	513	1767
Iridium	4086	3633	3201	15,129
Lithium	1.7	4.9	5.8	17.3

The supply levels for platinum, palladium, rhodium, ruthenium and iridium, as part of total supply of PGMs, were established based on historical data published by Johnson Matthey (2013a) (platinum, 46%; palladium, 43%; rhodium, 5%; ruthenium, 5%; iridium, 1%). These proportions can, of course, change, as only platinum, palladium and rhodium are currently used in autocatalysts and supply of these metals will grow at higher rate than of iridium and ruthenium. However, this assumption was made based on the fact that most PGMs are mined together and production of platinum and palladium currently determines maximum production of the others. Furthermore, ruthenium also has the potential to be used in catalysts, particularly in fuel cells (Albers et al. 2008).

Regardless of the scenarios considered, PGMs have hundreds to thousands times higher surplus cost values than lithium. However, once compared with the current production costs, the differences between PGMs and lithium are not that significant and they vary depending on the scenario considered. For example, surplus costs for ruthenium are about 37 (scenario 1), 33 (scenario 2) and 29% (scenario 3) of the current production costs. These proportions for lithium work conversely to those for ruthenium and are as follow: 10 (scenario 1), 28 (scenario 2) and 34% (scenario 3) of the current production costs. These results indicate that problematic price increases of lithium are unlikely if the latest technological trends in the automotive sector will continue up to 2070. Surplus costs for ruthenium are approximately one-third of the current production costs in all scenarios; hence, a threat of their price increases by 2070 will largely depend on the discovery of new deposits and the ability of new technologies to push these costs down over time. This also applies to lithium if the increasing electrification of road transport will continue up to 2070.

## Discussion

Evaluation of environmental impacts in the context of LCIA is always a choice of both midpoint and endpoint measures (Hauschid et al. 2013; Jasinski et al. 2016). Measures at the midpoint level are indicators placed at the location in the impact pathway and the endpoint modelling measures the severity of the damage that is modelled by the midpoint indicator (Hauschild et al. 2013). Existing estimates of surplus cost for metals are based on midpoint-to-endpoint modelling by linking the ore grade decrease function with the increasing marginal extraction costs of resources (Goedkoop et al. 2008). However, to date, an exact causal relationship between ore grade decrease and surplus costs has not been established (Vieira et al. 2016). Ponsioen et al. (2014), in the LC-Impact project, estimated surplus cost for fossil fuels without midpoint-to-endpoint modelling. They confirmed the suggestions made by Jolliet et al. (2004) that in some cases of modelling, at the endpoint level, the pathway could be better modelled without the inclusion of an indicator at the midpoint level. This study proves that minerals other than fossils (e.g. metals) can be modelled without an ore grade decrease function, something that in any case has been heavily criticised by authoritative bodies (Drielsma et al. 2016).

Existing studies into surplus cost confirmed that the major difficulty in estimating a surplus cost indicator relates to the acquisition of data for the production cost of minerals. These costs were either assumed constant across all mines (Goedkoop et al. 2008), or provided by authoritative institutions, such as the IEA (Ponsioen et al. 2014), or purchased from commercial databases (Vieira et al. 2016). This study showed that it is possible to collect own data and create original cost-cumulative availability curves in order to calculate a surplus cost indicator. The construction of these curves is both time and resource intensive as it requires browsing data from a number of different sources. For example, a master’s student spent approximately 1 year on the collection of data for lithium with the assistance of various industry and government officials, historical documents and studies (Yaksic and Tilton, 2009). The data collection process for PGMs was less intense and it took approximately 3 months, between January and March 2016. The quality of data collected is comparable to data from expert-driven consulting services. For instance, the average surplus cost values derived from the LC-Impact (Vieira et al. 2016) and this study represent a good fit with factor differences of 1.04, 1.85 and 0.62 for platinum, palladium and rhodium, respectively. The LC-Impact is based on real mining cost data purchased from the commercial database World Mine Cost Data Exchange. It would be both unexpected and surprising if the data were to match perfectly as the two methods are based on slightly different assumptions and have different data coverage.

Cost-cumulative availability curves, on which a surplus cost indicator is based, constructed for PGMs and lithium reflect the availability of minerals only from known deposits under current conditions (this is, current technology, prevailing labour and other input prices, and so on). Ideally, these curves should concern all known and unknown deposits as well as their current and future production costs (Yaksic and Tilton 2009). This, however, is rarely the case in practice as reliable information on unknown deposits and future technological developments is not available. A single attempt to construct this curve based on both known and unknown deposits can be found in Aguilera et al. (2009). According to Yaksic and Tilton (2009), this does not pose a serious problem as long as one keeps in mind that both new discoveries and the cost-reducing effect of new technology are likely to shift these curves down and to the right over time. It needs to be recognised that cost-cumulative curves are not fixed, and they have tendency to move around with each periodic update of the curve (Humphreys 2013; Drielsma et al. 2016). Hence, it is likely that new blocks will be continuously added to cost-cumulative availability curves for PGMs and lithium between now and 2070 as geological knowledge and extraction technology improves. This, in turn, will have an impact on surplus cost estimates for these metals, which currently do not incorporate any information about technological change over time and entry of new high-quality mines to the market. It is expected that new discoveries and technologies will shift surplus costs down in the future. Hence, the results provided in this paper can be interpreted as the upper cost limits (the worst-case possibilities), which are likely to be decreasing with the discovery of new deposits and cost-reducing technologies. The surplus cost estimates can be easily updated if new data become available in the future.

Considering the fact that cost-cumulative availability curves for PGMs and lithium (and resultant surplus cost estimates for these metals) capture only a small part of the total available resources and utilise the current production costs data, the results should not be interpreted as an indication of availability and potential scarcity of these resources in the long-run. Tilton and Lagos (2007) and Humphreys (2013) explained that the long-run mineral costs and prices are far more reliable warning indicators of future resource scarcity or lack of availability. This, however, is both a limitation and a strength of this work. Modelling these costs and prices in the long-run may suffer from huge uncertainties and significant inconsistencies by trying to anticipate something that nobody can know much (Humphreys, 2013). These uncertainties were avoided in this study by providing a mid-term outlook for the real threat of PGMs and lithium scarcity and the potential economic implications of their depletion from now up to 2070. Drielsma et al. (2016) recommended that existing cost-cumulative availability curves are the most suitable to analyse individual minerals in the 30–100-year time frame.

## Conclusions

This study has developed the life cycle impact assessment characterisation factors for platinum group metals and lithium to 2070 based on the surplus cost indicator. The surplus cost was calculated for three different scenario projections for future mineral production considering future market dynamics, recyclability rates, demand-side innovation and technological developments (particularly in the automotive sector) and economic growth. Surplus cost estimates (in US dollars per kilogramme in the year 2014) ranged from US$ 6545–8354 for platinum, US$ 3583–4573 for palladium, US$ 8281–10,569 for rhodium, US$ 513–655 for ruthenium, US$ 3201–4086 for iridium and US$ 1.70–5.80 for lithium.

This study sheds a useful view on whether depletion of PGMs and lithium could have any serious economic consequences from now up to 2070 under different production scenarios. Compared with the current production costs, the results indicate that problematic price increases of lithium are unlikely if the latest technological trends in the automotive sector will continue up to 2070. Surplus costs for PGMs are approximately one-third of the current production costs in all scenarios; hence, a threat of their price increases by 2070 will largely depend on the discovery of new deposits and the ability of new technologies to push these costs down over time. This also applies to lithium if the increasing electrification of road transport will continue up to 2070.

Surplus cost estimates for PGMs and lithium are based on cost-cumulative availability curves, which at the moment, capture only a small part of the total available resources (only known deposits) and utilise the current production costs data. Hence, the results of this study should be interpreted in the short- to mid-term (from now up to 2070) and not as an indication of availability and potential scarcity of these resources in the long-run (post 2070). Modelling and incorporating unknown deposits and potential future mineral production costs into these curves is the subject of future work. However, one needs to bear in mind that modelling these variables in the long-run may suffer from huge uncertainties and significant inconsistencies by trying to anticipate something that nobody can know much.

Regardless of these limitations, this study provides useful insight into the availability of PGMs and lithium up to 2070. It provides information about geological and economic risks of minerals depletion in the short-term and thus help to address these risks in the most optimal manner. It also proves that if time and resources permit, reliable surplus cost estimates can be calculated, at least in the short-run, based on the construction of one’s own curves with the level of quality comparable to expert-driven consulting services. The results are not fixed and should be periodically updated if new data become available between now and 2070.

## Electronic supplementary material

ESM 1 (DOCX 185 kb)

## Abbreviations

ADP: Abiotic resource depletion potential
EV: Electric vehicle
FCV: Fuel cell vehicle
HDV: Heavy-duty vehicle
HV: Hybrid vehicle
IEA: International Energy Agency
LCA: Life cycle assessment
LCIA: Life cycle impact assessment
LDV: Light-duty vehicle
MCI: Marginal cost increase
MDV: Medium-duty vehicle
PHEV: Plug-in hybrid electric vehicle
