# Supplementary material for: The life cycle impact for platinum group metals and lithium to 2070 via surplus cost potential
Source: Int J Life Cycle Assess. 2017 May 11;23(4):773–86. doi: 10.1007/s11367-017-1329-4 (PMC6566217; doi:10.1007/s11367-017-1329-4)
Supplement: Supplementary file 1 — (DOCX 185 kb) [file 11367_2017_1329_MOESM1_ESM.docx]

electronic supplementary material

[Environmental LCC](javascript:popupClassificationDetail(53))

**The life cycle impact for platinum group metals and lithium to 2070 via surplus cost potential**

**Dominik Jasiński^1^ • James Meredith^2^ • Kerry Kirwan^1^**

Received: 27 September 2016 / Accepted: 21 April 2017

© Springer-Verlag Berlin Heidelberg 2017

Responsible editor: Andrea J. Russell-Vaccari

^1^ Warwick Manufacturing Group, University of Warwick, Coventry CV4 7AL, UK

^2^ Department of Mechanical Engineering, University of Sheffield, Sheffield S1 3JD, UK

🖂 Dominik Jasinski

[d.jasinski@warwick.ac.uk](mailto:d.jasinski@warwick.ac.uk)

**Contents**

The process of a cost-cumulative availability curve for PGMs 1

List of PGM mines and projects and available PGM resources 10

PGM deposits and their production costs used to construct the PGMs cost-cumulative availability curve 19

Lithium deposits and their production costs used to construct the lithium cost-cumulative availability curve 21

**The process of constructing a cost-cumulative availability curve for PGMs**

A cost-cumulative availability curve for PGMs had to be developed in order to estimate the marginal cost increase and thus surplus costs for these minerals. The development process is presented in Fig. S1.

**Fig. S1** The research process used to construct cost cumulative availability curves for the PGMs

Each step in this process is explained in the following subsections.

*Data collection process*

The construction of a cost-cumulative availability curve requires the gathering of geological and production cost data ([Tilton 2003](#_ENREF_93)), which is both time and resource intensive as it requires browsing data from a number of different sources. The data collection process for PGMs was thus divided into three phases:

- **Phase 1** involved analysis of the distribution of PGM deposits around the globe. The best data for Phase 1 were collected from the USGS ([USGS 2014](#_ENREF_96)), British Geological Survey ([BGS 2009](#_ENREF_10)), Geoscience Australia ([Hoatson et al. 2014](#_ENREF_43)), Natural Resources Canada ([NRC 2015](#_ENREF_73)), Geological Survey of Finland (online) and [Johnson Matthey (2013)](#_ENREF_52). Based on these data, a global map of major PGM deposits was created.
- **Phase 2** involved analysis of sectoral information in order to identify PGM mining companies in each country with PGM deposits. For example, the Department of Mineral Resources in the Republic of South Africa released a report detailing all of the PGM mines operating within the country ([Moumakwa 2014](#_ENREF_68)). Details of Australian PGM exploration and mining projects are given on the official website of Geoscience Australia. Furthermore, the International Platinum Group Metals Association publishes information on major PGM producers. Based on this process, a list of PGM mines and producers was developed for each country, taking into account that not all PGM deposits are yet being mined. For instance, the Duluth Complex in the USA, Artic Platinum project in Finland and Kalplats Platinum project in South Africa remain in the processes of exploration, feasibility study or mining development. Several PGM mines have been closed over the last five years due to unfavourable market conditions and declining PGM prices ([Johnson Matthey 2013](#_ENREF_52)).
- **Phase 3** involved analysis of annual, technical and production reports, press releases, investor presentations, feasibility studies and the official websites of PGM mining, exploration and consulting companies. These sources of data were complemented, where necessary, with the governmental publications from Phases 1 and 2. Data on deposit type, mine type, ore grade, total resources, production volumes, operational and capital costs were collected for PGM mines and deposits.

The data collection process took approximately three months, between January and March 2016. Relevant PGM deposits and projects not included in this study are a deposit in Kambalda Province (Australia), deposits in Russia (Kondyor, Koryak and Central Urals), the Arctic Platinum Project (Penikat Complex in Finland) and the dunite pipes in South Africa, which are now largely mined out ([Scoon 2009](#_ENREF_84)). Data for these deposits were not available but they account for no more than 1 % of the total availability of PGMs. The most relevant PGM deposits with regard to the availability of PGMs are the Bushvald Complex in South Africa, Great Dyke province in Zimbabwe and deposits in Russia, accounting for nearly 94 % of all available PGMs.

The next step was to estimate the geological composition and production costs for each PGM mine, project or deposit based on the data collected.

*Estimating the geological composition of PGMs*

A typical deposit contains various metals but there is usually a main metal that justifies the exploration of a given deposit ([Vieira et al. 2016](#_ENREF_100)). PGMs are mined as both the main and accompanying metals of nickel (Ni) and copper (Cu) deposits (see Fig. S2).

In any case, mining companies tend to report the total amount of proven and probable reserves and measured and indicated resources of the deposit, along with the average ore grades (grams per tonne, or total ounces in the case of PGMs) of the main and accompanying minerals in the deposit (see, for example, [Anglo American 2014](#_ENREF_6); [African Rainbow Minerals 2015](#_ENREF_3)). PGM reserves and resources are usually aggregated into a single value for either six elements (6E = platinum, palladium, rhodium, ruthenium, iridium and gold), four elements (4E = platinum, palladium, rhodium and gold) or three or less elements (usually platinum, palladium and gold) ([USGS 2014](#_ENREF_96)).


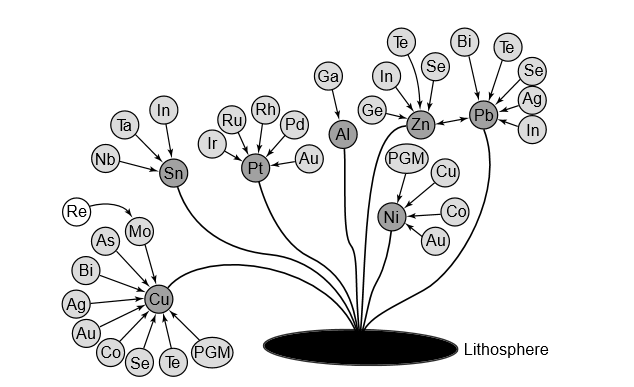


**Fig. S2** Base metals and accompanying minor metals produced as by-products ([source: Hagelüken and Meskers 2010](#_ENREF_38))

The ore grades per element for each deposit would need to be known in order to estimate the exact amount of each PGM element available worldwide, which was not the case in this study. Instead, a general concentration of PGM elements was assumed based on the existing literature ([Theart and De Nooy 2001](#_ENREF_92); [Crundwell et al. 2011](#_ENREF_16); [Zientek et al. 2014](#_ENREF_107)) for the following deposits: Merensky, UG2, Platreef (South Africa), Magnetite reefs and Ni-Cu sulphide. The concentrations of PGMs for other reefs in the USA, Canada, Finland, Greenland and Australia were sourced from the websites and reports of mining and exploration companies such as Stillwater Mining Company, Platina Resources Limited and North America Palladium. The generalised concentration of PGM elements in different deposit types is summarised in Table S1.

**Table S1** The general concentration of PGM elements identified in different deposit types

|  | Merensky (g/t) | UG2 (g/t) | Platreef  (South Africa) (g/t) | Ni-Cu Sulphide  (g/t) | Magnetite reefs  (g/t) | Stillwater (J-M Reef) (g/t) | Platreef (Canada/Finland/Greenland) (g/t) | Reefs (Australia) (g/t) |
| --- | --- | --- | --- | --- | --- | --- | --- | --- |
| Platinum | 3.3 | 2.5 | 1.3 | 1.7 | 0.65 | 0.12 | 0.275 | 1.1 |
| Palladium | 1.4 | 2 | 1.4 | 4.29 | 0.73 | 0.45 | 1.865 | 1.5 |
| Rhodium | 0.2 | 0.5 | 0.09 | 0.21 | - | 0.005 | - | 0.1 |
| Ruthenium | 0.4 | 0.7 | 0.12 | - | - | - | - | - |
| Iridium | 0.1 | 0.1 | 0.02 | - | - | - | - | - |
| Gold | 0.2 | 0.02 | 0.1 | 0.19 | 0.04 | - | 0.14 | - |

*Estimating production costs of PGM deposits*

The second element of the cost-cumulative availability curve, following on from geological composition, is to estimate the production costs per deposit, technology or country. Data on the production costs associated with minerals are generally not available to the public and are therefore difficult to obtain ([Yaksic and Tilton 2009](#_ENREF_105)). However, some mining and exploration companies publish their results or feasibility studies for the benefit of potential investors. The process of estimating production costs for PGM deposits comprised four steps:

- **Step 1:** annual operating costs (also called cash costs) and production volumes were collected from a variety of sources (see the data collection section, Phase 3). A typical cash cost usually includes on-mine, processing, administration and sometimes smelting and refining costs ([Implats 2015](#_ENREF_48)). These costs do not include taxation and royalties. Data were collected from the last five years of operations. Several mines operating in South Africa were affected by a series of strikes in 2014 ([see Anglo American 2014](#_ENREF_6)), meaning operating costs for that year were exceptionally high. Production costs from 2014 for all affected mines were not considered in this study as it was a one-off situation which did not reflect the real cost of mineral production. In the case of scoping and feasibility studies, production costs were usually provided in the form of different mining scenarios ([see Murahwi et al. 2010](#_ENREF_70)).
- **Step 2:** capital expenditures for mineral production were collected. The estimation of capital expenditures on an annual basis is more complicated than for operational costs since capital expenditures are incurred largely at the start of mining, with additional irregular expenditures incurred in subsequent years of running the project. If ultimate capital expenditures for the lifetime of the project are not known, the annual distribution of capital costs is best reflected by the depreciation of buildings and equipment ([Aguilera et al. 2009](#_ENREF_4)). This approach was also adopted in this study.
- **Step 3:** total cost per year was calculated by adding together operating and capital costs. At this stage, this cost was usually estimated either for the total joint mineral output per mine or, at best, for the joint output of PGMs and gold. In either case, an accounting technique of joint and by-product costing had to be applied in order to allocate total production costs to specific mining outputs. [Bhimani et al. (2008)](#_ENREF_11) and [Drury (2013)](#_ENREF_20) distinguished two major categories of methods for the allocation of joint costs: one based on physical measures (such as weight and volume) and the other based on sales values (relative to the market values of the respective products). The physical measures method assumes that the cost per unit is the same for each product, in contrast to the sales value method which allocates a higher proportion of joint costs to products with higher sales values ([Drury 2013](#_ENREF_20)). The second method is more in line with the opportunity cost concept and was therefore used in this study. It better represents society’s perception of how much they are willing to give up in order to produce a given mineral. For example, people are prepared to pay more for extracting platinum than they are for extracting ruthenium and this difference is not evident when using the physical measures method. Hence, the total production cost per mineral (*TPC_x_*) was calculated from:

$${TPC}_{x}=TPC*\frac{(V_{x}*P_{x})}{TR}$$

where *TPC* is the total cost of the joint mineral production output, *V_x_* is the output of a given mineral, *P_x_* is the market price of a given mineral and *TR* is the total revenue derived from the output of the joint mineral production. The mineral prices and currency exchange rates (if production costs were reported in currencies other than $US) used for calculating *TPC_x_* are provided in Table S2.

- **Step 4:** the total annual cost per unit produced was calculated by dividing *TPC_x_* by *V_x_* for a given year. Unit costs for most mines considered on-mine, milling and processing costs only. However, a few companies included the smelting and refining stages in their accounts. Unit costs for these mining companies were reduced by 18 %, which represents the approximate proportion of smelting and refining costs in the total mineral production cost (own calculations based on annual reports from Anglo American Platinum, Lonmin Platinum, Northam Platinum and Impala Platinum). All unit costs were adjusted for inflation using the CPI inflation calculator available at the Bureau of Labor Statistics website and converted into US dollars for the year 2014 ($US_2014_).

**Table S2** Annual average prices and currency exchange rates used to estimate mineral production costs

| **Mineral** | **2010** | **2011** | **2012** | **2013** | **2014** | **2015** |
| --- | --- | --- | --- | --- | --- | --- |
| Platinum ($US/t oz) | 1,614.58 | 1,720.13 | 1,554.57 | 1,490.19 | 1,389.51 | 1,060.49 |
| Palladium ($US/t oz) | 528.35 | 736.02 | 646.52 | 726.86 | 809.62 | 721.77 |
| Rhodium ($US/t oz) | 2,456.28 | 2,000.6 | 1,292.75 | 1,067.18 | 1,172.05 | 1136 |
| Ruthenium ($US/t oz) | 197.11 | 165.86 | 112.16 | 75.44 | 64.85 | 47.63 |
| Iridium ($US/t oz) | 639.47 | 1,034.73 | 1,134.06 | 825.95 | 555.28 | 543.6 |
| Gold ($US/t oz) | 1,228 | 1572 | 1,673 | 1,415 | 1,269 | 1170 |
| Nickel ($US/tonne) | 21,804 | 22,890 | 17,533 | 15,018 | 16,863 | 12,635 |
| Copper ($US/tonne) | 7,533.12 | 8,813.99 | 7,949.79 | 7,325.89 | 6,858.51 | 5,952.42 |
| Cobalt ($US/tonne) | 43,320.39 | 35,295.65 | 28,792.1 | 27,028.4 | 30,864.4 | 28,880.26 |
| Chromium ($US/tonne) |  | 27 | 20 | 19 | 18 | 17 |
| Silver ($US/t oz) | 19 | 24 | 31 | 35 | 19 | 17 |
| Lithium ($US/tonne) | 5,180 | 5,180 | 6,060 | 6,800 | 6,690 | 6,400 |

Note: Data sourced from the USGS and Johnson Matthey’s websites

| **World Bank data** |  |  |  |  |  |  |
| --- | --- | --- | --- | --- | --- | --- |
| **Exchange rates** | **2010** | **2011** | **2012** | **2013** | **2014** | **2015** |
| South Africa ZAR/$US | 7.32 | 7.26 | 8.21 | 9.66 | 10.85 | 12.15 |
| Canada $CAN/$US | 0.97 | 0.99 | 1 | 0.97 | 0.89 | 0.78 |

*Shaping the cost-cumulative availability curve for PGMs*

Following the estimation of geological distribution and production costs, the cost-cumulative availability curve for PGM deposits could then be constructed. The curve was constructed following a process similar to that used by [Yaksic and Tilton (2009)](#_ENREF_105). For each deposit, a minimum and maximum production cost was selected based on the calculated total costs per unit produced. This selection of minima and maxima enabled the dynamics and uncertainties associated with the potential fluctuation of PGM production costs in the future to be captured. It also allowed for the inclusion of other projects (deposits) in the cost-cumulative availability curve for which production costs could not be calculated, assuming they had the potential to be mined within the cost range estimated for other deposits in the country that had similar geological configurations, geographical locations and socio-economic situations. The cost-cumulative availability curve was created by ordering PGM deposits based on their minimum production costs, from lowest to highest, and adding together the amount of PGMs available within each deposit.

**Table S3** List of PGM mines and projects and available PGM resources

| **Country** | **Company** | **Province** | **Mine/project** | **Deposit type** | **Mine type** | **Grades (g/t)** | **Total resources (tonnes)** | **PGM resources (kg)** |
| --- | --- | --- | --- | --- | --- | --- | --- | --- |
| Australia | Platinum Australia/Panoramic Resources LtD | East Kimberley | Panton | Merensky | Project | 1.07–2.83 | 14,300,000 | 69,927 |
| Australia | Platina Resources Limited | Munni Munni Complex | Munni Munni | Merensky | Project on hold | 1.1–1.6 | 23,600,000 | 68,428 |
| Australia | Platina Resources Limited | New South Wales | Owandale Scandium Project | Dunite type (Sc + Pt) | Project (open pit) | 0.36–0.53 | 23,700,000 | 8,616 |
| Botswana | Tati Nickel/Norilsk Nickel | Selkirk | Selkirk mine | Nickel-copper sulphide | Open pit and underground | 0.56–0.57 | 135,300,000 | 76,981 |
| Canada | Pacific North West Capital | East Bull Lake | The River Valley Project | Platreef | Project | 0.79–0.88 | 127,250,000 | 89,080 |
| Canada | Stillwater | Port Coldwell Alkalic Complex (Ontario) | Marathon | Platreef | Project (open pit) | 0.41–1.09 | 121,000,000 | 121,335 |
| Canada | Lonmin Platinum/Vale | Sudbury Complex (Meteorite type) | Denison | Nickel-copper sulphide | Project | 1.9–2.3 | 380,000 | 2,227 |
| Canada | Glencore | Sudbury Complex (Meteorite type) | Sudbury | Nickel-copper sulphide | Underground | 1.9­–2.4 | 47,500,000 | 83,046 |
| Canada | Vale | Sudbury Complex (Meteorite type) | Sudbury | Nickel-copper sulphide | Underground | 1.9–2.5 | 85,200,000 | 187,430 |
| Canada | Glencore | Ungava Belt | Raglan | Nickel-copper sulphide | Underground | 1.7–2.52 | 35,500,000 | 113,839 |
| Canada | North America Palladium | Wabigoon Greenstone Belt | Lac Des Iles | Platreef | Open pit and underground | 2.08–2.87 | 86,507,000 | 141,303 |
| Canada | n/a | Thompson Belt | n/a | Nickel-copper sulphide | Being explored | 0.69–0.83 | 154,000,000 | 127,835 |
| China | Jinchuan Group International Resources | Jinchuan province | n/a | Nickel-copper sulphide | n/a | 0.2–0.26 | 515,000,000 | 133,745 |
| Finland | First Quantum Minerals Ltd | Lapland Region | Kevitsa | Platreef | Open pit | 1.82–2.2 | 272,200,000 | 97,976 |
| Finland | Arctic Platinum | Portimo Complex | Suhanko, Narkaus, Penikat | Platreef | Project (open pit) | 0.36–1.47 | 208,000,000 | 405,745 |
| Greenland | Platina Resources Limited | East Coast | Skaergaard | Pt+Pd+Au | Project (underground) | 0.65–2.07 | 202,220,000 | 291,129 |
| Russia | Norilsk Nickel | Kola Division (Pechenga deposit) | Kola Peninsula mines | Nickel-copper sulphide | Open pit and underground | 0.07–5.57 | 632,481,000 | 45,847 |
| Russia | Amur Minerals | Kun-Manie province | Ikenskoe, Soboloevsky, Maly Kurumkon, Vodorazdelny | Nickel-copper sulphide | Project | 0.1–0.3 | 120,800,000 | 24,199 |
| Russia | Norilsk Nickel | Polar Division | Talnakh mines | Nickel-copper sulphide | Open pit and underground | 0.07–5.58 | 2,806,500,000 | 14,401,594 |
| South Africa | African Rainbow Minerals/Impala | Bushveld Complex - Eastern Limb | Two Rivers Platinum + Tamboti | UG2 and Merensky | Underground | 3.11–6.19 | 165,100,000 | 721,601 |
| South Africa | XK Platinum Partnership/Anglo/Glencore | Bushveld Complex - Eastern Limb | Mototolo | UG2 | Underground | 3.17–3.33 | 32,400,000 | 133,745 |
| South Africa | African Rainbow Minerals/Anglo | Bushveld Complex - Eastern Limb | Modikwa Platinum | UG2 and Merensky | Open pit and underground | 2.78–7.21 | 256,830,000 | 1,231,698 |
| South Africa | Lonmin Platinum | Bushveld Complex - Eastern Limb | Limpopo (including 50 % Dwaalkop) | UG2 and Merensky | Underground | 3.46–4.37 | 174,800,000 | 706,049 |
| South Africa | Northam Platinum | Bushveld Complex - Eastern Limb | Booysendal (North, South and North mine) | UG2 and Merensky | Underground | 3.23–5.05 | 820,570,000 | 3,182,819 |
| South Africa | Northam Platinum | Bushveld Complex - Eastern Limb | Zondereinde | UG2 and Merensky | Underground | 5.08–7.35 | 421,580,000 | 2,521,559 |
| South Africa | Northam Platinum | Bushveld Complex - Eastern Limb | Everest (acquired from Aquarius) | UG2 | Underground | 3.33 | 29,030,000 | 96,732 |
| South Africa | Anglo American Platinum | Bushveld Complex - Eastern Limb | Twickenham Platinum (Next to Modikwa and Marula) | UG2 and Merensky | Underground | 3.47–4.20 | 647,000,000 | 3,586,231 |
| South Africa | Northam Platinum/Lonmin | Bushveld Complex - Eastern Limb | Dwaalkop (50 %) | UG2 and Merensky | Project | 2.98–4.35 | 75,610,000 | 277,132 |
| South Africa | Aquarius Platinum | Bushveld Complex - Eastern Limb | Millennium, Vygenhoek, Hoogland, Vygehoek (Everest province) | UG 2 | Project | 2.88–5.11 | 42,700,000 | 369,198 |
| South Africa | Aquarius Platinum | Bushveld Complex - Eastern Limb | Zondernaam and Hoedspruit (Limpopo province) | UG2 and Merensky | Project | 5.00–7.98 | 109,980,000 | 674634 |
| South Africa | Atlatsa Resources/Anglo | Bushveld Complex - Eastern Limb | Bokoni Platinum (including Ga-Phasha) | UG2 and Merensky | Open pit and underground | 4.45–6.78 | 833,600,000 | 4,789,935 |
| South Africa | Impala Platinum | Bushveld Complex - Eastern Limb | Marula Platinum | UG2 and Merensky | Underground | 3.85–7.38 | 107,700,000 | 805,580 |
| South Africa | African Rainbow Minerals/Impala | Bushveld Complex - Eastern Limb | Crocodile River Mine | UG2 | Long-term maintenance | 3.92–4.05 | 41,000,000 | 162,827 |
| South Africa | Eastern Platinum Limited/Anglo | Bushveld Complex - Eastern Limb | Kennedy’s Vale | UG2 and Merensky | Long-term maintenance | 2.56–4.95 | 436,610,000 | 1,600,585 |
| South Africa | Eastern Platinum Limited/Anglo | Bushveld Complex - Eastern Limb | Mareesburg | UG2 | Long-term maintenance | 2.19–5.26 | 15,800,000 | 62,207 |
| South Africa | Platinum Australia/African Thunder Platinum Ltd | Bushveld Complex - Eastern Limb | Smokey Hills (Limpopo province) | UG2 | Long-term maintenance | 6.6 | 5,500,000 | 24,883 |
| South Africa | Aquarius Platinum | Bushveld Complex - Eastern Limb | BLUE Ridge (Limpopo province) | UG2 | Long-term maintenance | 3.28 | 46,180,000 | 151,163 |
| South Africa | Anglo American Platinum | Bushveld Complex - Northern Limb | Mogalakwena Platinum | Platreef | Open pit | 2.60–3.03 | 3,696,200,000 | 8,758,739 |
| South Africa | Lonmin Platinum | Bushveld Complex - Northern Limb | Akanani | Platreef | Project | 3.9 | 293,706,000 | 1,145,535 |
| South Africa | Ivanhoe Mines | Bushveld Complex - Northern Limb | Ivanhoe project | Platreef | Project | 2 | 852,000,000 | 2,946,121 |
| South Africa | Platinum Group Metals | Bushveld Complex - Northern Limb | Waterberg Project | UG2 and Merensky | Project | 3.01–3.95 | 286,880,000 | 902,001 |
| South Africa | Impala Platinum | Bushveld Complex - Western Limb | Impala Platinum mine | UG2 and Merensky | Open pit and underground | 3.76–7.37 | 435,000,000 | 3,402,720 |
| South Africa | Tharisa Minerals | Bushveld Complex - Western Limb | Tharisa | UG2 | Open pit | 1.56 | 828,000,000 | 1,293,283 |
| South Africa | Royal Bafokeng Platinum/Anglo | Bushveld Complex - Western Limb | Bafokeng-Rasimone Platinum (33 % Anglo) | UG2 and Merensky | Underground | 5.11–7.38 | 323,349,600 | 1,978,613 |
| South Africa | Lonmin Platinum | Bushveld Complex - Western Limb | Lonmin Marikana | UG2 and Merensky | Open pit and underground | 4.84–4.97 | 875,796,000 | 4,305,157 |
| South Africa | Sedibelo Platinum Mines | Bushveld Complex - Western Limb | Pilanesberg Platinum (Shared activities with Northam) | UG2 and Merensky | Open pit | 1.41–1.61 | 1,032,000,000 | 1,558,284 |
| South Africa | Wesizwe/ Platinum Group Metals Limited | Bushveld Complex - Western Limb | Bakubung (including Maseve projects, Pilanesberg province) | UG2 and Merensky | Project | 3.67–6.26 | 153,324,000 | 765,736 |
| South Africa | Aquarius Platinum/Anglo | Bushveld Complex - Western Limb | Kroondal mine | UG2 | Underground | 3.53–3.80 | 24,200,000 | 143,076 |
| South Africa | Anglo American Platinum | Bushveld Complex - Western Limb | Tumela | UG2 and Merensky | Underground | 4.14–6.63 | 479,400,000 | 2,737,106 |
| South Africa | Anglo American Platinum | Bushveld Complex - Western Limb | Bathopele | UG2 and Merensky | Underground | 2.85–3.02 | 49,900,000 | 174,179 |
| South Africa | Anglo American Platinum | Bushveld Complex - Western Limb | Dishaba | UG2 and Merensky | Underground | 3.85–5.59 | 188,500,000 | 1,069,960 |
| South Africa | Anglo American Platinum | Bushveld Complex - Western Limb | Siphumelele (Khomanani) | UG2 and Merensky | Underground | 3.28–5.37 | 263,100,000 | 1,371,663 |
| South Africa | Anglo American Platinum | Bushveld Complex - Western Limb | Union mine | UG2 and Merensky | Underground | 3.98–6.55 | 260,300,000 | 1,486,746 |
| South Africa | Anglo American Platinum | Bushveld Complex - Western Limb | Thembelani and Khuseleka | UG2 and Merensky | Underground | 3.61–5.02 | 238,700,000 | 1,209,925 |
| South Africa | Glencore | Bushveld Complex - Western Limb | Eland Platinum (including Zilkaatsnek and Schietfontein) | UG2 | Long-term maintenance | 3.1–4.5 | 180,533,000 | 685,832 |
| South Africa | Aquarius Platinum/Anglo | Bushveld Complex - Western Limb | Marikana (next to Kroondal) | UG2 | Long-term maintenance | n/a | 31,800,000 | 158,628 |
| South Africa | Lonmin Platinum/Anglo | Bushveld Complex - Western Limb | Pandora | UG2 | Underground | 4.65 | 154600,000 | 715,380 |
| South Africa | African Rainbow Minerals/Anglo/Platinum Australia | Kalplats Deposit | Kalahari Platinum Project | Magnetite reefs | Project | 1.34–3.24 | 69,910,000 | 103,575 |
| South Africa | African Rainbow Minerals/Norilsk | Uitkomst Complex | Nkomati (Uitkomst Complex) | Nickel-copper sulphide | Open pit and underground | 0.65–0.92 | 226,590,000 | 217,724 |
| USA | Duluth Metals | Duluth Complex | Twim Metals Minnesota Project | Nickel-copper sulphide | Project (feasibility assessment) | 0.22–0.75 | 4,000,000,000 | 2,637,575 |
| USA | Stillwater | Stillwater Complex Montanta | Stillwater mine (including Blitz project) | Merensky (J-M Reef) | Underground | 0.4–0.59 | 15,970,000 | 283,322 |
| USA | Stillwater | Stillwater Complex Montanta | East Boulder mine (including Graham Creek project) | Merensky (J-M Reef) | Underground | 0.4–0.59 | 32,928,000 | 408,140 |
| Zimbabwe | Impala Platinum | Great Dyke | Zimplats (Ngezi and Hartley) | UG2 and Merensky | Underground | 3.6 | 2,060,400,000 | 7,411,959 |
| Zimbabwe | Impala Platinum/Aquarius | Great Dyke | Mimosa | UG2 and Merensky | Underground | 3.17–3.86 | 129,200,000 | 470,907 |
| Zimbabwe | Anglo American Platinum (Amplats) | Great Dyke | Unki Platinum | UG2 and Merensky | Underground | 3.10–3.64 | 254,700,000 | 1,038,856 |

**Table S4** PGM deposits and their production costs used to construct the PGMs cost-cumulative availability curve

| **Country** | **Location** | **Deposit type** | **Grades** | **PGM resources (kilograms)** | **Min cost ($US/t oz)** | **Max cost ($US/t oz)** |
| --- | --- | --- | --- | --- | --- | --- |
| Russia | Kola Division (Pechenga deposit) | Nickel-copper sulphide | 0.07–5.58 | 44,480 | 250 | 520 |
| Russia | Kun-Manie province | Nickel-copper sulphide | 0.1–0.3 | 23,479 | 250 | 520 |
| Russia | Polar Division | Nickel-copper sulphide | 0.07–5.58 | 13,973,380 | 250 | 520 |
| China | Jinchuan province | Nickel-copper sulphide | 0.2–0.26 | 129,770 | 280 | 470 |
| Canada | Sudbury Complex | Nickel-copper sulphide | 1.9–2.3 | 264,590 | 310 | 650 |
| Canada | Thompson Belt | Nickel-copper sulphide | 0.69–0.83 | 124,040 | 310 | 650 |
| Canada | Ungava Belt (Raglan mine) | Nickel-copper sulphide | 1.7–2.52 | 110,460 | 310 | 650 |
| USA | Duluth Complex | Nickel-copper sulphide | 0.22–0.75 | 2,559,150 | 310 | 650 |
| Australia | Fifield in New South Wales (Owandale Project) | Dunite type (Sc + Pt) | 0.36–0.53 | 8,616 | 320 | 480 |
| Canada | East Bull Lake | Platreef | 0.79–0.88 | 83,611 | 360 | 690 |
| Canada | Lac Des Iles | Platreef | 2.08–2.87 | 132,630 | 360 | 690 |
| Canada | Port Coldwell Alkalic Complex (Stillwater Marathon) | Platreef | 0.41–1.09 | 113,890 | 360 | 690 |
| Botswana | Selkirk | Nickel-copper sulphide | 0.56–0.57 | 74,690 | 380 | 790 |
| South Africa | Uitkomst Complex | Nickel-copper sulphide | 0.65–0.92 | 211,250 | 380 | 790 |
| USA | Stillwater Complex Montanta | Merensky (J-M Reef type) | 0.4–0.59 | 691,460 | 460 | 540 |
| Zimbabwe | Great Dyke | UG2 and Merensky | 3.1–3.86 | 8,747,077 | 460 | 960 |
| South Africa | Bushveld Complex - Northern Limb | Platreef | 2–3.9 | 12,426,290 | 520 | 660 |
| South Africa | Bushveld Complex - Eastern Limb | UG2 and Merensky | 2.19–7.98 | 20,685,570 | 520 | 1140 |
| South Africa | Bushveld Complex - Western Limb | UG2 and Merensky | 1.41–7.38 | 22,604,950 | 520 | 1140 |
| South Africa | Bushveld Complex - Northern Limb | UG2 and Merensky | 3.01–3.95 | 884,340 | 520 | 1140 |
| South Africa | Kaplats Deposit | Magnetite Reefs | 1.34–3.24 | 100,660 | 550 | 600 |
| Finland | Lapland Region | Platreef | 0.31–0.39 | 91,960 | 570 | 890 |
| Finland | Portimo Complex | Platreef | 1.82–2.2 | 380,840 | 570 | 890 |
| Greenland | Skaergaard Province | Platreef | 0.65–2.07 | 273,260 | 570 | 890 |
| Australia | East Kimberley (Panton) | Merensky | 1.07–2.83 | 69,930 | 1,010 | 1,100 |
| Australia | Munni Munni Complex | Merensky | 1.1–1.6 | 68,430 | 1,010 | 1,100 |

**Table S5** Lithium deposits and their production costs used to construct the lithium cost-cumulative availability curve ([source: Yaksic and Tilton, 2009](#_ENREF_105))

| **Country** | **Location** | **Deposit type** | **Grades (g/t)** | **Recoverable lithium (tonnes)** | **Min production cost ($US/kg of lithium)** | **Min production cost ($US/kg of lithium)** |
| --- | --- | --- | --- | --- | --- | --- |
| Chile | Atacama | In brines | 0.15 | 16,065,000 | 9.0 | 12.9 |
| China (Tibet) | DXC | In brines | 0.04–0.05 | 63,270 | 12.9 | 15.5 |
| China (Tibet) | Zhabuye | In brines | 0.05–0.1 | 688,500 | 12.9 | 15.5 |
| China | Taijinaier | In brines | 0.3 | 117,000 | 14.2 | 16.8 |
| Argentina | Hombre Muerto | In brines | 0.06 | 366,750 | 14.2 | 16.8 |
| Argentina | Olaroz | In brines | 0.09 | 146,250 | 14.2 | 16.8 |
| USA | Silver Peak | In brines | 0.023 | 18,000 | 14.2 | 16.8 |
| Argentina | Rincon | In brines | 0.04 | 841,500 | 15.5 | 19.4 |
| Chile | Maricunga | In brines | 0.092 | 99,000 | 15.5 | 19.4 |
| Australia | Greenbushes | Minerals | 1.36 | 127,500 | 15.5 | 19.4 |
| Bolivia | Uyuni | In brines | 0.04 | 2,475,000 | 15.5 | 23.2 |
| Zimbabwe | Masvingo | Minerals | 1.4 | 28,350 | 15.5 | 23.2 |
| Canada | Bernic Lake | Minerals | 1.28 | 9,300 | 15.5 | 23.2 |
| USA | Cherryville | Minerals | 0.68 | 167,500 | 15.5 | 23.2 |
| Portugal | Barroso-Alvao and Covas de Barroso | Minerals | 0.37–0.77 | 5,000 | 18.1 | 25.8 |
| China | Gajika | Minerals | n/d | 280,000 | 18.1 | 25.8 |
| China | Maerkang | Minerals | n/d | 110,000 | 18.1 | 25.8 |
| Brazil | Brazil | Minerals | n/d | 42,500 | 19.4 | 25.8 |
| Canada | Separation Rapids | Minerals | 0.62 | 36,100 | 23.2 | 28.4 |
| Canada | Quebec | Minerals | 0.53 | 53,000 | 24.5 | 28.4 |
| China | Jaijika | Minerals | 0.59 | 225,000 | 24.5 | 28.4 |
| China | Qaidam Basin | In brines | n/d | 909,000 | 19.4 | 32.3 |
| USA | Searies Lake | In brines | 0.0065 | 14,220 | 25.8 | 32.3 |
| USA | Kings Mountain | Minerals | 0.69 | 100,000 | 25.8 | 32.3 |
| Russia | Etykinskoe | Minerals | 0.23–0.79 | 290,000 | 27.1 | 34.9 |
| Namibia | Namibia | Minerals | n/d | 5,750 | 28.4 | 36.1 |
| USA | Salton Sea | In brines | 0.022 | 450,000 | 28.4 | 36.1 |
| USA | Great Salt Lake | In brines | 0.004 | 236,700 | 28.4 | 36.1 |
| Israel-Jordan | Dead Sea | In brines | 0.002 | 900,000 | 31.0 | 38.7 |
| Zaire | Manono-Kitololo | Minerals | 0.58 | 1,150,000 | 31.0 | 38.7 |
| Mali | Bougouni Area | Minerals | 1.4 | 13,000 | 31.0 | 38.7 |
| Canada | Yellowknife | Minerals | 0.66 | 64,500 | 32.3 | 38.7 |
| USA | McDermitt | Hectorites | 0.24–0.53 | 450,000 | 45.2 | 60.7 |
| USA | North Carolina | Minerals | n/d | 1,300,000 | 45.2 | 60.7 |
| Russia | Russian pegmatites | Minerals | n/d | 290,000 | 45.2 | 60.7 |
| USA | Smackover | In brines | 0.0386 | 450,000 | 64.5 | 82.6 |
| n/a | Oceans | Seawater | 0.000017 | 44,800,000,000 | 90.4 | 129.1 |

**References:**

African Rainbow Minerals (2015) Annual Integrated Report 2015

Aguilera RF, Eggert RG, Lagos CG, Tilton JE (2009) Depletion and the future availability of petroleum resources. The Energy Journal**,** pp 141-174

Anglo American (2014) Operations Review 2014

BGS (2009) Platinum. London: Natural Environment Research Council

Bhimani, A., Horngren, C. T. & Foster, G. 2008. Management and cost accounting, Pearson Education.

Crundwell FK, Moats M, Ramachandran V (2011) Extractive metallurgy of nickel, cobalt and platinum group metals, Elsevier

Drury CM (2013) Management and cost accounting. Springer, Andover

Hagelüken C, Meskers CE (2010) Complex life cycles of precious and special metals. Linkages of sustainability, p4

Hoatson DM, Miezitis Y, Jaireth S, Huston DL (2014) Platinum-group elements in Australia. Geological setting, mineral systems, and potential. Geoscience Australia - Department of Industry

Implats (2015) Annual Financial Statement 2015

Jarvis M, Lange G-M, Hamilton K, Desai D, Fraumeni B, Edens B, Ferreira S, Li H, Chakraborti L, Kingsmill W (2011) The changing wealth of nations: measuring sustainable development in the new millennium

Jasinski D, Meredith J, Kirwan K (2015) A comprehensive review of full cost accounting methods and their applicability to the automotive industry. J Clean Prod 108**:**1123-1139

Johnson Matthey (2013) Platinum 2013. Royston, United Kingdom: Johnson Matthey

Moumakwa D (2014) Operating Platinum Group Metal Mines in South Africa, 2014. South Africa - Department of Mineral Resources

Murahwi C, Shoemaker S, Gowans R, Lemieux J, Jacobs C (2010) Technical Report on the Updated Feasibility Study for the Marathon PGM-Cu Project. Marathon, Ontario, Canada**,** pp 43-101

Natural Resources Canada (NRC) (2015) Minerals and Metals Fact Book - 2015. Ministry of Natural Resources

Scoon RN (2009) Discovery and geology of the platinum group element deposits of the Bushveld Complex, South Africa. Society of Economic Geologists Newsletter 78**:**13-19

Theart H, De Nooy C (2001) The platinum group minerals in two parts of the massive sulphide body of the Uitkomst Complex, Mpumalanga, South Africa. South African J Geol 104**:**287-300

Tilton JE (2003) On Borrowed Times? Assessing the Threat or Mineral Depletion, Washington, Resources for the Future

US Geological Survey (USGS) (2014) 2014 Minerals Yearbook - Platinum-Group Metals. Reston: U.S. Geological Survey, Department of the Interior

Van der Heijden K (2011) Scenarios: the art of strategic conversation. John Wiley & Sons

Van Oers L, De Konng A, Guineé JB, Huppes G(2002) Abiotic resource depletion in LCA. Road and Hydraulic Engineering Institute, Ministry of Transport and Water, Amsterdam

Vieira M, PonsioenTC, Goedkoop MJ, Huijbregts MA (2016) Surplus Cost Potential as a Life Cycle Impact Indicator for Metal Extraction. Resources, 5**,** 2

Yaksic A, Tilton JE (2009) Using the cumulative availability curve to assess the threat of mineral depletion: The case of lithium. Resources Policy 34**:**185-194

Zientek ML, Causey JD, Parks HL, Miller RJ (2014) Platinum-group elements in southern Africa: mineral inventory and an assessment of undiscovered mineral resources: Chapter Q in Global mineral resource assessment. US Geological Survey
